# Supplementary material for: Splice-Junction-Based Mapping of Alternative Isoforms in the Human Proteome
Source: Cell Rep. Author manuscript; Available in PMC 2020 Jan 15. (PMC6961840; doi:10.1016/j.celrep.2019.11.026)
Supplement: 3 [file NIHMS1546469-supplement-3.zip › DF2/PXD0010154/Pancreas-76-Q9Y3S1-GQGSSTSSLAPGPEPGPQPALH.pdf]

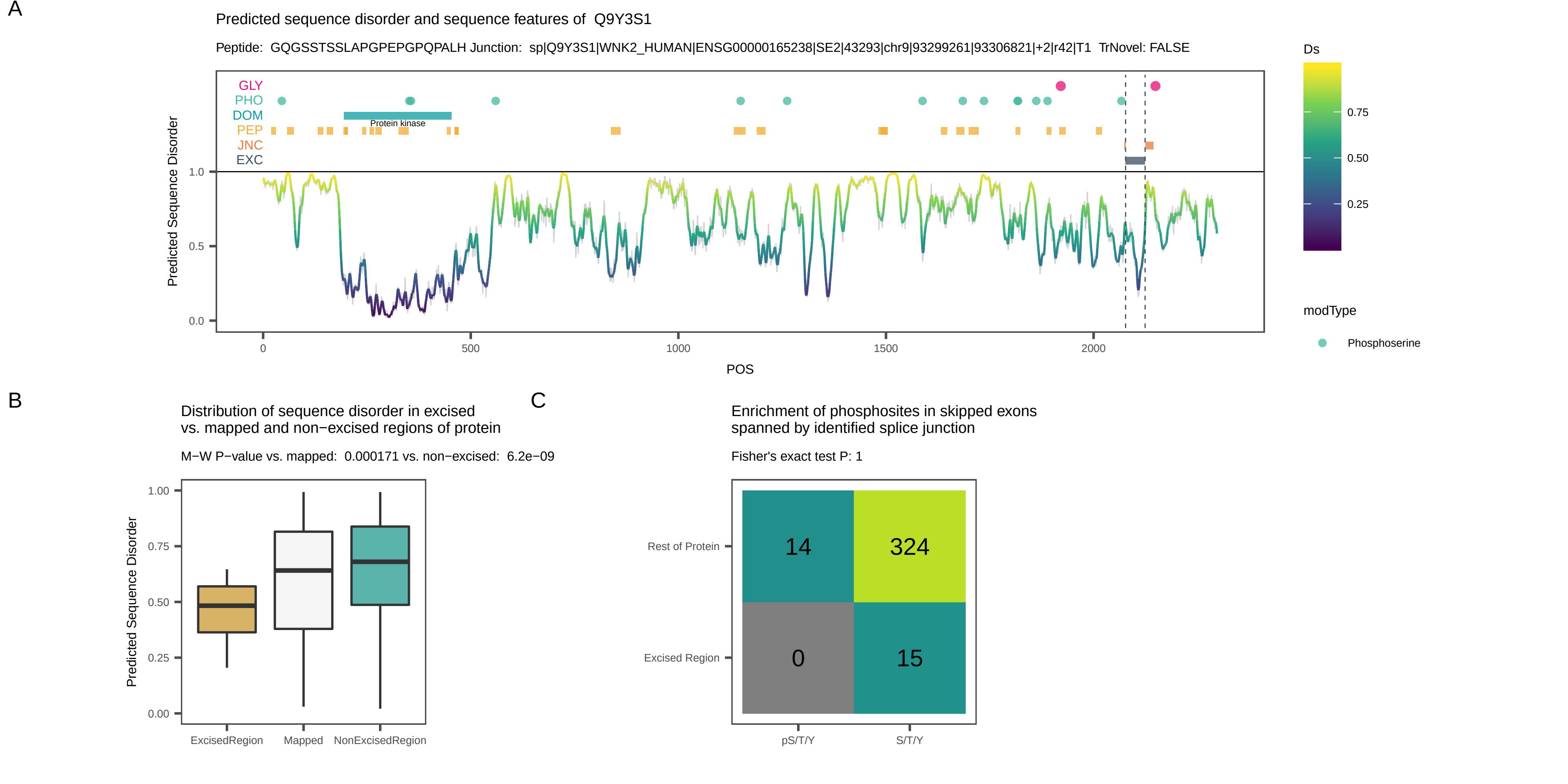
A
Predicted sequence disorder and sequence features of Q9Y3S1
Peptide: GQGSSTSSLAPGPEPGPQPALH Junction: sp|Q9Y3S1|WNK2_HUMAN|ENSG00000165238|SE2|43293|chr9|93299261|93306821|+2|r42|T1 TrNovel: FALSE
GLY
PHO
DOM
PEP
JNC
EXC
Protein kinase
Predicted Sequence Disorder
1.0
0.5
0.0
0
500
1000
1500
2000
POS
Ds
0.75
0.50
0.25
modType
Phosphoserine
B
Distribution of sequence disorder in excised
vs. mapped and non−excised regions of protein
M−W P−value vs. mapped: 0.000171 vs. non−excised: 6.2e−09
Predicted Sequence Disorder
1.00
0.75
0.50
0.25
0.00
ExcisedRegion
Mapped
NonExcisedRegion
C
Enrichment of phosphosites in skipped exons
spanned by identified splice junction
Fisher's exact test P: 1
Rest of Protein
Excised Region
14
324
0
15
pS/T/Y
S/T/Y
